# Natural History of the Neotropical Arboreal Ant, *Odontomachus hastatus*: Nest Sites, Foraging Schedule, and Diet

**DOI:** 10.1673/031.012.4801

**Published:** 2012-04-10

**Authors:** Rafael X. Camargo, Paulo S. Oliveira

**Affiliations:** ^1^ Departamento de Biologia Animal, Universidade Estadual de Campinas, C.P. 6109, 13083-970 Campinas SP, Brazil; ^2^ Biology Department, University of Ottawa, 30 Marie Curie, Ottawa ON, KIN 6N5 Canada

**Keywords:** activity rhythms, Atlantic forest, Brazil, bromeliad-nesting ants, canopy-dwelling ants, Formicidae, nocturnal ants, Ponerinae

## Abstract

The ecology of most arboreal ants remains poorly documented because of the difficulty in accessing ant nests and foragers in the forest canopy. This study documents the nesting and foraging ecology of a large (∼13 mm total length) arboreal trap—jaw ant, *Odontomachus hastatus* (Fabricius) (Hymenoptera: Formicidae) in a sandy plain forest on Cardoso Island, off the coast of Southeast Brazil. The results showed that *O. hastatus* nested in root clusters of epiphytic bromeliads, most commonly *Vriesea procera* (70% of nest plants). Mature *O. hastatus* colonies include one to several queens and about 500 workers. Foraging by *O. hastatus* is primarily nocturnal year—round, with increased foraging activity during the wet/warm season. The foragers hunt singly in the trees, preying on a variety of canopy—dwelling arthropods, with flies, moths, ants, and spiders accounting for > 60% of the prey captured. Although predators often have impacts on prey populations, the ecological importance of *O. hastatus* remains to be studied.

## Introduction

Ants are dominant social insects that occur in a wide variety of habitats, and exhibit a vast diversity of nesting and feeding habits (Wheeler 1910). Ant foraging strategies may range from solitary hunting to different levels of cooperative foraging mediated by recruitment behavior among nestmates (Hölldobler and Wilson 1990). The majority of ant species are considered generalists by feeding on a broad range of animal— and plant—derived food items, but numerous species may have specialized diets (Carroll and Janzen 1973; Hölldobler and Wilson 1990). Ants are especially dominant in tropical habitats where they are remarkably diverse both on the ground and on vegetation (Brown 2000). Surprisingly, however, very little is known about the natural history and ecology of most tropical ants. Even for large and conspicuous species there is a general lack of information on their basic ecological features such as nest sites, activity rhythms, foraging substrate, and diet.

Most species in the subfamily Ponerine are regarded as predators because they possess powerful mandibles and are armed with a sting (Hölldobler and Wilson 1990). Ponerine species may have diverse feeding habits and foraging modes, ranging from solitary to group hunting, both on the ground and/or foliage (Peeters and Crewe 1987; Brown 2000). Foraging strategies may consist of active hunting for live prey, scavenging for dead arthropods, gathering of plant and/or insect exudates, pearl bodies, and fleshy fruits and seeds (e.g., Duncan and Crewe 1994; Déjean and Suzzoni 1997; Blüthgen et al. 2003; Oliveira and Freitas 2004; Dutra et al. 2006).

The ponerine genus *Odontomachus* is widely distributed in tropical and warm temperate environments and is especially abundant in the Neotropical region where numerous species may occur from semi—arid environments to rain forests (Brown 2000). *Odontomachus* ants are well—known by their trap—jaws that are used to capture and kill prey (Spagna et al. 2008). Individual foragers usually hunt on a broad variety of invertebrates (e.g., Déjean and Bashingwa 1985; Ehmer and Hölldobler 1995; Raimundo et al. 2009), but may also consume small vertebrates (Facure and Giaretta 2009), plant and insect exudates (Blüthgen et al. 2003; Souza and Francini 2010), and nutrient—rich fleshy fruits (Passos and Oliveira 2002, 2004). Because visual access to arboreal ant foragers in the three dimensional forest canopy is inherently difficult, detailed studies on the foraging ecology of tropical ponerines have focused mostly on ground—dwelling species whose laden workers are easier to follow and their prey identified (but see for instance Dejean and Suzzoni 1997; Djieto-Lordon et al. 2001).

*Odontomachus hastatus* (Fabricius) (Hymenoptera: Formicidae) is a poorly— studied arboreal species inhabiting tropical rainforests of Central and South America (Brown 1976; Gibernau et al. 2007). In Southeast Brazil, the species is facultatively polygynous and commonly nests among roots of epiphytic bromeliads in coastal sandy forests (Oliveira et al. 2011). This study provides qualitative and quantitative data on the natural history and ecology of *O. hastatus* with emphasis on nest sites, daily and seasonal activity schedules, and diet in a forest reserve on Cardoso Island off the coast of Brazil.

## Materials and Methods

### Study area

Fieldwork was undertaken from August 1999 to October 2001 in the sandy plain forest of the State Park of Cardoso Island (22500 ha, 0– 800 m a.s.l.), located off the coast of São Paulo State, Southeast Brazil (25° 03′ S, 47° 53′ W). The vegetation presents an open canopy formed by 5–15 m—tall trees growing on poor sandy soil, and abundant terrestrial and epiphytic bromeliads. The climate is characterized by two main seasons: a cool and less rainy period (winter) from April to August (minimum temperature 13 °C, rainfall ∼500 mm), and a warm and rainy period (summer) from September to March (maximum 32 °C, rainfall up to 2600 mm) (Funari et al. 1987).

### Nest sites

**Figure 1.**
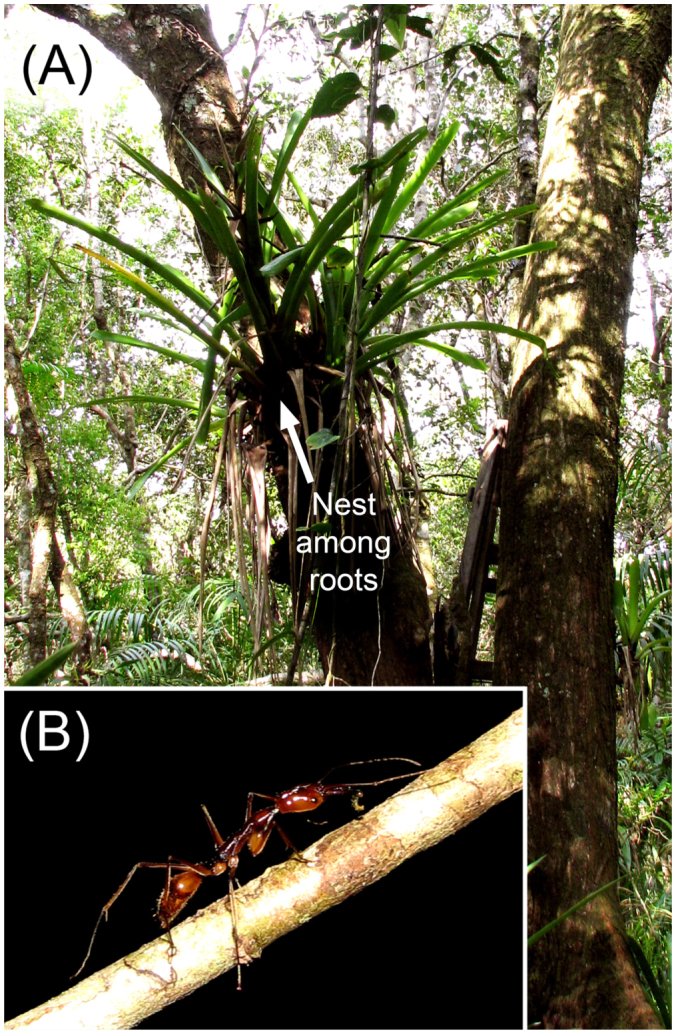
(A) General view of two clumped epiphytic bromeliads (*Vriesia*) in the sandy forest of Cardoso Island, southeast Brazil; *Odontomachus hastatus* uses the epiphytes' root mass as nesting site for the colony. (B) Returning forager of *Odontomachus hastatus* with a recently—captured termite in the mandibles. Photographs courtesy of Pedro Rodrigues. High quality figures are available online.

The density of *O. hastatus* colonies in the sandy forest at Cardoso Island was estimated by surveying epiphytic bromeliads within a plot of 13,350 m^2^. Locations of ant—occupied plants were found by following loaded workers attracted to sardine baits distributed on vegetation. 45 epiphytic bromeliads were found hosting *O. hastatus* colonies within root clusters (Figure 1). The plant species used as nest (single or clumped epiphytes) and the height of the nest relative to the ground were recorded for each colony. The size and composition of *O. hastatus* colonies (i.e., number of workers, queens, and presence of brood) was described in the field by collecting 19 entire nest bromeliads and carefully counting the ants living within the root clusters. Additional details on colony demography and colony organization in *O. hastatus* is provided elsewhere (Oliveira et al. 2011).

### Activity rhythm and diet

Foraging activity of each of two tagged colonies of *O. hastatus* was evaluated once per season (each colony on a different day) in the dry/cold (August 2000) and wet/warm (February 2001) periods. The activity rhythm of the colonies was monitored by recording all ants entering or exiting the nests during the first 40 min of every two hours for a period of 24 hours. Nocturnal observations were carried out using a flashlight covered with a red filter to reduce disturbance of the ants. Air temperature was recorded simultaneously with ant samplings.

The food items retrieved by *O. hastatus* were surveyed by removing them from the mandibles of returning foragers. Collections were performed in February and March 2001 during the period of greatest foraging activity (from 17:30 to 23:00). The food items taken from foragers were preserved in 70% alcohol and brought to the laboratory for identification. The items were then kept in an oven at 70 °C for 3 hours to determine their dry weights with a Mettler H51Ar analytical balance (Mettler—Toledo International Inc., www.mt.com). In the few cases where the collection of the item was not possible, the identification of the food was included in the survey. To avoid disturbance of ant foragers, no food item was collected during sessions monitoring the daily activity rhythm of ant colonies.

## Results

Collected colonies of *O. hastatus* contained 35 to 536 workers (291.2 ± 163.0 workers; N = 19 colonies), and approximately half of the colonies had more than one dealated queen (4.0 ± 3.7 queens; range 1–12; n = 18 queenright colonies). 45 nests were found in the 13,350 m^2^ sandy forest study plot (density of ∼33.7 nests/ha). All colonies nested among root clusters of single or clumped epiphytic bromeliads 1.5 to 4.6 m above ground (2.2 ± 1.2 m; N = 45) (see Figure 1). The species of Bromeliaceae most frequently used by *O. hastatus* for nesting were *Vriesea procera* (73%; 33/45 nests), followed by *Aechmea* spp. (13%), and *Quesnelia arvensis* and *Vriesea* sp. (7% each). The ants used *V. procera* opportunistically, since this plant species accounted for 80% of the epiphytic bromeliads sampled in the study area (Oliveira et al. 2011).

Individual foragers of *O. hastatus* searched for food usually in the canopy of the tree hosting the nest bromeliad. Hunters frequently used climbing lianas as bridges to go from tree to tree in the forest canopy, or to search for prey on lower plants (Figure 1). *O. hastatus* was never observed searching for food on the ground.

**Figure 2.**
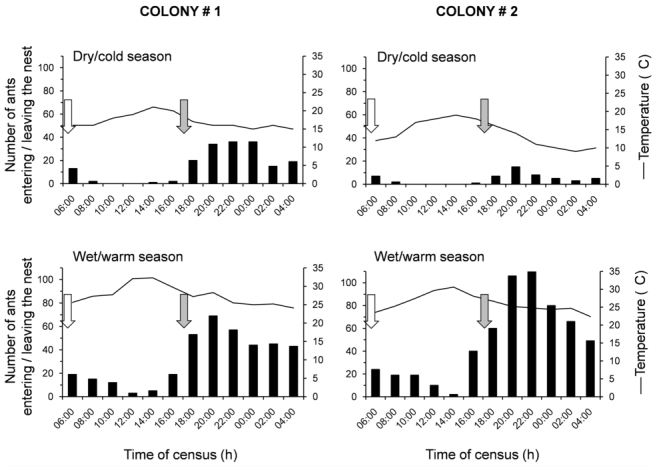
Foraging activity of *Odontomachus hastatus* colonies in the dry/cold (August) and wet/warm season (February), on Cardoso Island, southeast Brazil. Ant activity and air temperature were evaluated at two—hour intervals. The arrows indicate sunrise (white) and sunset (grey). High quality figures are available online.

Foraging activity typically begins at dusk around 17:30 and peaks near 20:00 (Figure 2). At sunset, individual foragers depart from nest bromeliads to hunt for prey among foliage. Typically as the first workers return with newly—captured prey, more ants tend to leave the nest. Foraging ceases at dawn between 06:00 and 08:00. The foraging rhythm is predominantly nocturnal throughout the whole year, with a marked increase in the overall worker activity in the wet/warm compared to the dry/cold season (Figure 2).

**Figure 3.**
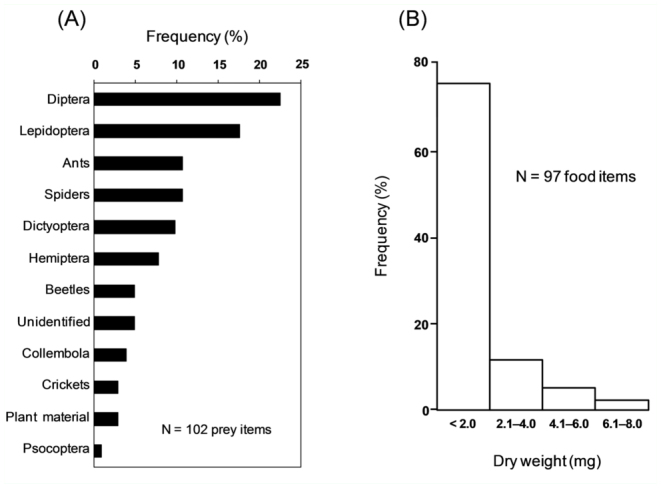
(A) Frequency distribution of different types of prey captured by *Odontomachus hastatus* foragers, and (B) of their dry weights, in the sandy forest of Cardoso Island, southeast Brazil. Data are based on collections from returning ants of two focal colonies, during the peak of their foraging activity (17:30 to 23:00). High quality figures are available online.

From a total of 102 food items registered as part of the diet of two colonies of *O. hastatus*, canopy—dwelling arthropods comprised the vast majority of the items retrieved by ant foragers in the study area (Figure 3A). The most representative prey groups were dipterans (adults), lepidopterans (larvae and adults), ants (workers and winged forms), and spiders, which together accounted for over 60% of the prey captured. *Odontomachus hastatus* is a typical generalist predator, with the vast majority of the prey consisting of organisms captured alive (88% of the identified animal items), most of which of dry weight < 2.0 mg (Figure 3B).

## Discussion

*Odontomachus* species have frequently been documented nesting in both terrestrial and epiphytic bromeliads (e.g., Davidson and Epstein 1989; Dejean and Olmsted 1997; Oliveira et al. 2011), and colonies have been recorded occupying variable parts of their nest plants, including the basket—like leaf structure, the root mass, or spaces between the epiphyte and the host tree (Dejean et al. 1995; Blüthgen et al. 2000; Oliveira et al. 2011). The nesting space used by *O. hastatus* apparently has a strong influence on the size and social structure of the colonies at Cardoso Island, since worker and queen numbers were shown to be positively associated with the diameter of the root mass housing the colony (Oliveira et al. 2011).

Foraging activity in *O. hastatus* is likely influenced by the photoperiod since the ants leave the nest bromeliads to hunt just after dusk and return before dawn, a daily activity pattern also reported for other arthropods, including ants (Rosengren 1977; Heinrich 1993; Machado et al. 2000; Raimundo et al. 2009). Indeed, arboreal *O. hastatus* remains primarily nocturnal year—round regardless of seasonal fluctuations in temperature, with a marked increase in foraging activity in the wet/warm season. A similar nocturnal rhythm was reported for the ground—dwelling species *O. chelifer* in a forest site in Southeast Brazil (Raimundo et al. 2009). Other ponerines, however, are known to alter their daily activity schedules to follow seasonal fluctuations in temperature and/or humidity (e.g., Déjean and Lachaud 1994; Medeiros and Oliveira 2009). For instance, in arid Australia, *Odontomachus* colonies have been reported to shift crepuscular activity in the spring toward nocturnal activity in the summer due to severe temperatures (Briese and Macauley 1980). Similarly, raids to termite nests by the Neotropical and obligate termitophagous species *Pachycondyla striata* change to the night period during the hot season (Leal and Oliveira 1995).

Seasonal fluctuations in the abundance of insect prey (Wolda 1988) and/or plant— derived resources such as extrafloral nectar, insect honeydew, and fleshy fruits (Rico-Gray and Oliveira 2007) may also affect foraging patterns and/or food preference by ant colonies year—round, and this is usually associated with the presence of larvae in the nest (i.e., Judd 2005). For instance, increased activity and foraging range by bromeliad—nesting *Gnampogenys moelleri* in the wet/warm season matches the period of increased quantity of brood in the colonies and greater availability of arthropod prey at Cardoso Island (Cogni and Oliveira 2004). Increased activity by *O. hastatus* foragers during the wet/warm season at Cardoso Island also corresponds with greater quantity of brood in the colonies (Oliveira et al. 2011).

Tank bromeliads are reservoirs of arthropod diversity, and some of the preferred prey groups consumed by *O. hastatus* (e.g., flies, ants, spiders) are among the most frequent inhabitants of bromeliad leaf baskets, or in their vicinity (see Richardson 1999; Frank et al. 2004; Gonçalves-Souza et al. 2010). This contrasts with other tropical generalist ground—dwelling *Odontomachus* spp., which hunt preferentially on termites (Fowler 1980; Déjean and Bashingwa 1985; Ehmer and Hölldobler 1995; Raimundo et al. 2009). As opposed to bromeliad—nesting *Gnamptogenys moelleri* that hunts chiefly within the nest plant (Cogni and Oliveira 2004), large *O. hastatus* is a typical generalist predator of canopy—dwelling arthropods that expands its hunting area up to 8 m from the nest plant, also using other epiphytes as foraging terrain (Rodrigues 2009). With the aid of their good vision (Oliveira and Hölldobler 1989) and efficient trap—jaws (Spagna et al. 2008), nocturnal *O. hastatus* hunters are able to capture fast—fleeing prey such as winged— insects (flies, moths, and ants) and spiders. Despite being regarded as mostly carnivorous, *Odontomachus* spp. also consume lipid— and protein—rich fleshy fruits on the ground of tropical forests and savannas (Passos and Oliveira 2002, 2004), as well as extrafloral nectar (Blüthgen et al. 2003) and insect honeydew (Souza and Francini 2010) on foliage. Since it was not possible to accurately follow *O. hastatus* foragers on the forest canopy at night, it is not known whether the ants consume plant or insect exudates. In the very few cases in which plant matter was brought to the nest (Figure 3), the food item was not identifiable. Although individual foragers of *O. hastatus* typically searched for food and retrieved prey without cooperation, an increase in forager departure as food is brought to the nest by early hunters may suggest an elementary form of recruitment, as noted for other species in this genus (Fowler 1980; Oliveira and Hölldobler 1989; Raimundo et al. 2009).

In conclusion, arboreal *O. hastatus* nests in root clusters of epiphytic bromeliads at Cardoso Island, with *Vriesea procera* accounting for nearly 70% of the nest plants recorded. Mature colonies contain nearly 500 workers and from one to several queens. The ant is primarily nocturnal year—round, with increased foraging activity during the wet/warm season, and a generalist diet consisting of a variety of canopy—dwelling arthropods. This study adds to the knowledge about the natural history and foraging ecology of tropical arboreal ponerines. Several avenues of investigation remain to be explored about the behavioral ecology of *O. hastatus*, most notably the modes of colony foundation, the ecological factors mediating polygyny in this species, the stability of epiphytic nest plants, as well as seasonal variation in diet and foraging range as related to food availability.
